# Asynchronous seasonal dynamics of nycteribiid bat flies and *Bartonella* spp. in Australian flying foxes (*Pteropus* spp.)

**DOI:** 10.1186/s13071-026-07243-1

**Published:** 2026-01-29

**Authors:** Brent D. Jones, Caylee A. Falvo, Chris Burwell, Tamika J. Lunn, Devin N. Jones-Slobodian, Evelyn Benson, Clifton D. McKee, Agnieszka Rynda-Apple, Raina K. Plowright, Daniel J. Becker, Kerry L. Clark, Hamish McCallum, Nicholas J. Clark, Alison J. Peel

**Affiliations:** 1 Centre for Planetary Health and Food Security, Griffith University, Nathan, QLD Australia; 2 Sydney School of Veterinary Science, University of Sydney, Camperdown, NSW Australia; 3 Department of Public and Ecosystem Health, College of Veterinary Medicine, Cornell University, Ithaca, NY USA; 4 Biodiversity and Geosciences, Queensland Museum, South Brisbane, QLD Australia; 5 Odum School of Ecology, University of Georgia, Athens, GA USA; 6 Center for the Ecology of Infectious Diseases, University of Georgia, Athens, GA USA; 7 Department of Microbiology & Cell Biology, Montana State University, Bozeman, MT USA; 8 Department of Epidemiology, Johns Hopkins Bloomberg School of Public Health, John Hopkins University, Baltimore, MD USA; 9 School of Biological Sciences, University of Oklahoma, Norman, OK USA; 10 Department of Public Health, University of North Florida, Jacksonville, FL USA; 11 School of Veterinary Science, University of Queensland, Gatton, QLD Australia; 12 Sydney Infectious Diseases Institute (Sydney ID), Faculty of Medicine and Health, University of Sydney, Camperdown, NSW Australia

**Keywords:** Bat flies, Nycteribiidae, *Cyclopodia*, Ectoparasite, Vector, *Bartonella*, *Borrelia*, Chiroptera, Pteropodidae, Flying fox, Host–parasite interaction, Vector transmission

## Abstract

**Background:**

Bat flies are ubiquitous ectoparasites of bats, recognised as potential vectors for viral and bacterial transmission between individual bats within a roost. Despite this, little is known about the seasonal dynamics of bat flies. Here, we present the results of a longitudinal study that compares seasonal prevalence and host risk factors for bat fly (Diptera: Nycteribiidae) parasitism with that of *Bartonella* and *Borrelia* spp. detected in *Pteropus alecto* and *P. poliocephalus* in eastern Australia.

**Methods:**

Flying foxes were sampled at nine different roosts in south-east Queensland and northern New South Wales between February 2018 and September 2022 using mist nets. Host and ectoparasite data were recorded, and bat fly specimens were collected for identification. Blood samples collected from the flying foxes were screened for the presence of *Bartonella* and *Borrelia* DNA using polymerase chain reaction (PCR).

**Results:**

Ectoparasite data were recorded from 2235 flying foxes and 840 had blood samples screened for *Bartonella* and *Borrelia* DNA. *Cyclopodia albertisii* was the predominate nycteribiid species identified, with few detections of *C. australis*. Nycteribiid prevalence had a consistent annual cycle (ranging from 8.6% to 100%) that depended on local climatic factors, increasing with increased temperature and humidity during summer and decreasing in winter. *Bartonella* spp. prevalence exhibited less variation seasonally (ranging from 50% to 100%) with a peak in winter that was driven by host age, with juvenile bats having a reduced probability of infection compared with subadults and adults. *Borrelia* spp. were rare and showed no clear seasonality.

**Conclusions:**

This study reports the longitudinal occurrence of the blood-borne bacteria *Bartonella* spp. and their likely ectoparasite vectors in Australian flying foxes. The findings contribute to knowledge of nycteribiid ecology critical for understanding their vector potential within flying fox roosts and provide direction for future research into nycteribiid-mediated transmission dynamics.

**Graphical Abstract:**

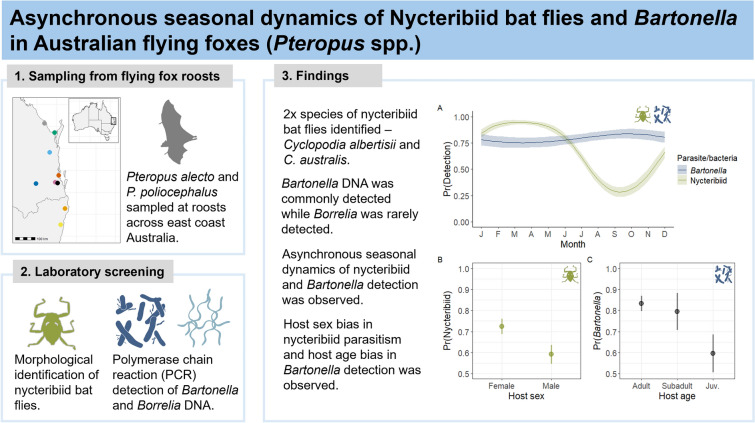

**Supplementary Information:**

The online version contains supplementary material available at 10.1186/s13071-026-07243-1.

## Background

A diverse range of ecological, behavioural, and immunological factors have been identified as contributing to the transmission of infectious agents from bat species to novel hosts (termed ‘spillover’). Overlapping spatial and temporal distributions, physiological stress resulting in dampening of immune function, novel host behaviour and susceptibility all interact in complex ways to influence pathogen shedding patterns and opportunities for cross-species transmission [1]. External parasites (ectoparasites) are recognised for their role as vectors of viruses and bacteria, and respond to both environmental and host factors. We define vectors as haematophagous arthropod hosts that contribute to the transmission of an infectious agent between allospecific or conspecific vertebrate hosts [2–4]. Although ectoparasites are frequently detected on bats, including true bugs (Hemiptera: Cimicidae and Polyctenidae), ticks (*Argas*, *Carios*, *Ixodes* and *Ornithodoros* species), fleas (Siphonaptera: Ischnopsyllidae and Macronyssidae), mites (Acari: Spinturnicidae, Rhinonyssidae, Macronyssidae and Myobiidae) and bat flies (Diptera: Streblidae, Nycteribiidae and Mystacinobiidae), their role in pathogen transmission within bat communities and from bat communities to other hosts remains underexplored [5].

Several blood-borne bacterial pathogens have been associated with bat ectoparasites and may serve as models for understanding broader parasite-mediated transmission dynamics. For example, *Borrelia* is a genus of spirochete bacteria that has a multi-host epidemiology involving wildlife species and arthropod vectors, most commonly ticks or lice [6]. Many *Borrelia* species cause zoonotic disease as well as disease in non-human animals including bats [7, 8]. Mounting evidence suggests bats may also function as wildlife hosts for *Borrelia* species, following detection in bats and ticks collected from bats [9–15]. *Bartonella* is a genus of intracellular Alphaproteobacteria known to colonise the red blood cells of many mammalian hosts and is also spread by haematophagous arthropods [16]. Again, many *Bartonella* species are recognised causes of zoonoses and also cause disease in non-human animals [17]. *Bartonella* species have been reported from bats and their ectoparasites globally, being detected in fleas, ticks, mites, bat bugs and bat flies [18].

Determining the potential of an arthropod as a vector requires consideration of their specialised life cycle and host associations. Bat flies have evolved a high degree of host specificity as obligate, haematophagous parasites of bats [19]. Nycteribiid bat flies are wingless and only able to survive off the host briefly; however, they do leave the host for copulation and larviposition in the roost environment [19, 20]. This presents an opportunity for horizontal transmission to occur if the bat flies return to a different host individual. The environmental pupal stage is the longest time nycteribiid bat flies are away from the host, when they spend approximately 3 weeks (up to one-third of the nycteribiid life span) in a puparium in the roost environment [19]. This unique lifestyle, combined with host roosting habit and social behaviours, has implications for the potential of bat flies to transmit other parasites or pathogens in a roost.

Australian flying foxes (*Pteropus* species) are the natural reservoir for three known zoonotic viruses: Australian bat lyssavirus (ABLV), Hendra virus (HeV) and Menangle virus (MenV) [21–23]. In contrast to the extensive research on viral dynamics in flying foxes, comparatively little is known about the nycteribiid bat flies they host. None of the studies exploring bat fly dynamics within the Australian context explicitly describe temporal dynamics of bat fly parasitism within flying foxes [24–26]. This lack of temporal data on bat fly parasitism represents a significant barrier to understanding potential vector-mediated pathways for pathogen transmission and how these might interact with other known ecological drivers of spillover events.

This study leverages the findings of a longitudinal, multi-roost survey of individual flying foxes in south-east Queensland and northern New South Wales to describe and compare the temporal prevalence dynamics of nycteribiids and blood-borne bacteria in flying fox roosts. We explore associations between host and abiotic variables and propose potential drivers of prevalence, thereby informing vector dynamics necessary for effective study of bat-borne viral and bacterial dynamics. Our study aims are to:

1. Describe the prevalence of nycteribiid bat flies, *Bartonella* spp. and *Borrelia* spp., in Australian flying fox roosts over time.
2. Identify potential factors related to the host or environment that contribute to the prevalence dynamics observed.

We predict that nycteribiid and *Bartonella* spp. will have similar prevalence dynamics and risk factors owing to the suspected vectorial nature of the relationship between them. We expect increases in the prevalence of nycteribiids will result in increased transmission between flying foxes as bat flies leave the host to reproduce, leading to a spike in prevalence of blood-borne bacteria as susceptible hosts introduced following birthing periods become infected with bacteria carried by the bat flies.

## Methods

### Field data collection

**Fig. 1:**
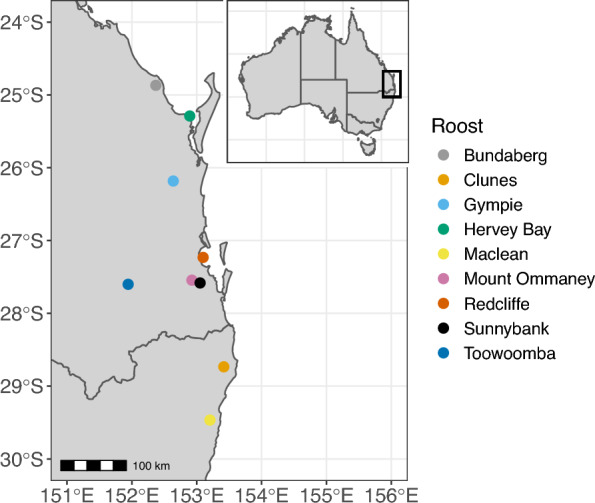
Map of roost locations across south-east Queensland and north-east New South Wales. Inset: The Australian continent with south-east Queensland (Qld) and north-east New South Wales (NSW) regions outlined

Field research was conducted under approvals from the Griffith University Animal Ethics Committee (certificate: ENV/10/16/AEC and ENV/07/20/AEC). Bat sampling sessions took place at nine different roost locations in south-east Queensland and north-east New South Wales between February 2018 and September 2022 (Fig. 1). Two locations, Toowoomba and Redcliffe, were longitudinally sampled sites while the other roosts were sampled opportunistically.

Capture and sampling protocols have been described previously [27, 28]. Briefly, individual flying foxes were caught prior to sunrise when returning to the roost using a mist net. The bats were immediately removed from the net upon capture and placed in individual holding bags while awaiting processing and release. All field staff received training prior to handling animals, and were wearing personal protective equipment (PPE), including eye protection, N95 respirators, clothing covering arms and legs, double sets of latex gloves and welding gloves, while directly handling conscious animals. All field staff had up-to-date protection against rabies virus, based on a full course of rabies vaccinations and adequate titre responses.

Bats were processed in the field under anaesthesia using isoflurane and oxygen. Biometric data were recorded, including species (black flying fox, *Pteropus alecto* or grey-headed flying fox, *Pteropus poliocephalus*), sex (male, female), weight (in grams), estimated age class (dependent pup, juvenile, subadult or adult as described in BatOneHealth [29]) and reproductive status. Briefly, dependent pups are still attached to their mothers and are approximately < 4 months of age; juveniles are volant, weigh < 450 g, have a forearm length < 150 mm, are non-reproductive (small nipples with no hair loss around them that would be suggestive of previously nursing a pup for females, and small penis with non-visible testicles for males), and considered to be young of that most recent birthing period (< 12 months of age); subadults are non-reproductive (females may have some hair loss starting around the nipple, males still have underdeveloped testicles), weigh between 450 and 550 g, have a forearm length approximately between 150 and 160 mm and are approximately between 12–24 months of age; adults are reproductively mature (recognised in females by larger nipples generally with hair loss around them, pregnancy or lactation, and in males by large penis and testicles and a musky odour), weigh > 550 g, have forearms longer than 160 mm when measured between the olecranon process of the ulna to the flexed carpus and are > 24 months of age [30]. The same criteria for each age class were used for both species of flying fox encountered in this study. Individuals were classified as non-reproductive (sexually immature), reproductive (either sexually mature male bats or sexually mature females that were not pregnant or lactating), pregnant (sexually mature females that had a palpable abdomen mass consistent with pregnancy) and/or lactating (sexually mature female bats that were lactating). Blood samples were collected from either the cephalic or uropatagial veins, and the presence of nycteribiid ectoparasites was noted, and a count of parasite intensity (0–10 and 10+) was recorded for most individuals. Counts were performed at the end the examination when both the dorsal and ventral aspects could be inspected and took no longer than a few seconds. The location of parasites on the host was not recorded. Parasites were collected in 70% ethanol in a subset of sessions (those between February 2018 and January 2019; *n* = 16 sessions) and stored at room temperature for identification. Each bat was administered subcutaneous fluids consisting of one part Baxter sodium chloride 0.45% and glucose 2.5% IV infusion (Baxter Healthcare, Australia) and one part Baxter Compound sodium lactate (Hartman’s Solution) (Baxter Healthcare, Australia) made up to a volume equal to 5% of body weight in millilitres to support recovery. Following recovery of anaesthesia, individuals were released back into the roost of capture.

### Parasite identification

Nycteribiids preserved in ethanol were submitted to the Queensland Museum for identification. Specimens were examined under Nikon SMZ18 binocular dissecting microscope and identified using external morphology. All nycteribiid specimens belonged to the genus *Cyclopodia* and were identified to species and sex using the keys in Maa [31] and by reference to identified specimens in the insect collection of the Queensland Museum.

### Blood-borne bacteria identification

Blood samples collected in the field were preserved on Whatman FTA cards until further processing. As outlined in Verrett et al. [15], DNA was extracted from the blood samples using Qiagen QIAamp DNA Investigator Kits (QIAGEN) and *Borrelia* screening was undertaken using polymerase chain reaction (PCR) targeting the 16S rRNA gene, flagellin (*flaB*) gene, and 16S–23S rRNA intergenic spacer (IGS). *Bartonella* detection was performed using nested PCR targeting the citrate synthase gene (*gltA*) as described in Crowley et al. [32].

### Environmental variables

Abiotic data were collected for each sampling session. Temperature, dewpoint temperature, solar radiation, precipitation, and evaporation data were collected using the ERA5 ECMWF atmospheric re-analysis of global climate accessed using Google Earth Engine [33, 34]. Briefly, each site was identified using geographical coordinates, and a buffer of a 10 km radius was used to create the shapefile for each. Image bands (representing an individual hour) for each variable were imported for the 28 day (~1 month) period preceding the final day of sampling for each session and were clipped to each site’s shapefile. The mean or total value for each image was determined (total value for precipitation and evaporation image bands) and exported in a csv file. This file contained the hourly mean or total for every hour of the 1-month period preceding each sampling session. Monthly summary measurements were thought most relevant for the current understanding of nycteribiid development and reproduction (lifecycle is 1 month in temperate region, cave-dwelling species [35]). Relative humidity was determined using temperature and dewpoint temperature results following the approach in Lawrence [36] using revised Magnus coefficients as recommended by Alduchov and Eskridge [37]:

$$RH=100 \times \left[\frac{{e}^{\frac{c \times {D}_{p}}{k + {D}_{p}}}}{{e}^{\frac{c \times T}{k+ T}}}\right]$$,

where $RH$ = relative humidity (%), ${D}_{p}$ = dewpoint temperature (C°), $T$ = temperature (C°), $c$ = 17.625 and $k$ = 243.04.

Oceanic Niño Index (ONI) data were retrieved from NOAA [38], including sea surface temperature and anomaly in the Niño 3.4 region in degrees Celsius. Given the ONI is taken as a 3-month rolling average, the value recorded for each session is taken to be the 3 months preceding the date the session took place.

### Statistical analysis

#### Description of data

All analyses were performed in RStudio (v2025.05.0 + 496) using the R programming language (v4.2.2) and tidyverse (v2.0.0) and MetBrewer (0.2.0) packages [39–42]. Exact 95% binomial confidence intervals for the prevalence of nycteribiids and blood-borne bacteria were determined in R, while median 95% confidence intervals were determined with the DescTools (v0.99.59) package [43] using the bootstrap method for individual intensity data. Right-truncated ectoparasite counts (10+) were converted to 11 to enable differentiation between individuals with an intensity of 10 and those with greater than 10, and comparison of parasite intensity among sessions. Four sessions showed potential ceiling effects, with either median (ACRED011) or Q3 values reaching this transformed maximum (ACBBG001, ACRED010, ACRED014).

Poulin’s index of discrepancy ($D$) was calculated to assess patterns of parasite aggregation according to the following equation:

$$D=1- \frac{2\sum_{i = 1}^{N}(\sum_{j=1}^{i}{x}_{j})}{\overline{x }N(N+1)}$$,

where $x$ is the number of parasites in host $j$ (when hosts are ranked from least to most parasitised) and $N$ is the total number of hosts [44]. *D* has a minimum value of 0 indicating no aggregation, i.e. all hosts harbour the same number of parasites, and a maximum value of 1 indicating complete aggregation, i.e. a single host harbours all parasites [44]. Given this measure is based on proportional ranking of both parasite and hosts, it should be more robust to right truncated data than alternative measures. Poulin’s *D* was calculated using ecopaR (v0.0.0.90) [45] in R.

#### Estimating seasonal and host risk factors of nycteribiid infestation and blood-borne bacterial infection

**Fig. 2:**
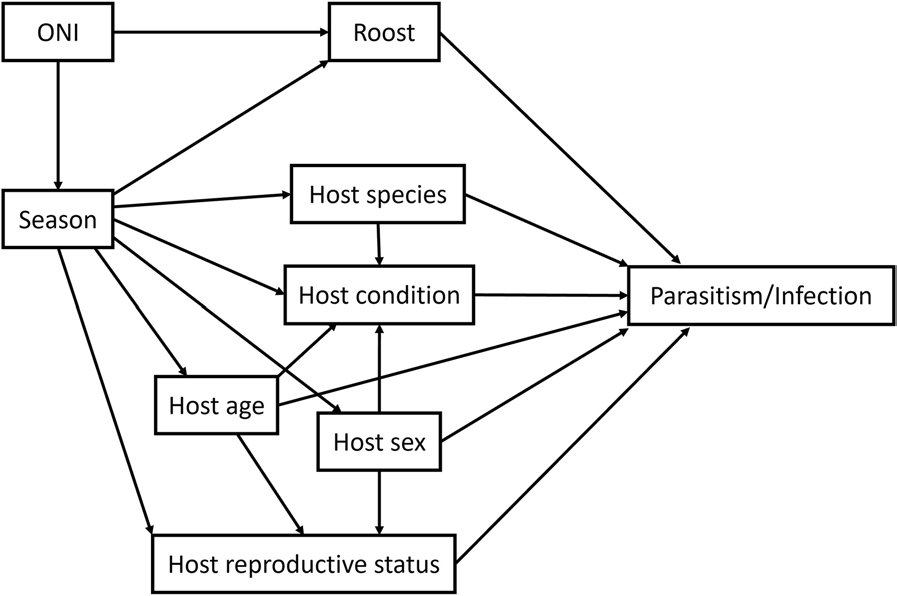
Directed acyclic graph (DAG) outlining assumptions for the parasite–host system. Variable selection for statistical models was informed by applying the backdoor criterion to identify the minimum adjustment set of variables required to reduce bias in the estimation of risk of infestation [47]. This is achieved by blocking paths that begin with an arrow into the variable of interest and end with an arrow into the outcome. For example, ‘season’ has a single arrow leading to it (ONI →  season), which also follows a path to the outcome (ONI → roost → nycteribiid parasitism). Successfully blocking this path requires inclusion of ONI as a covariate with ‘season’ in the model estimating the effect of season on the risk of nycteribiid infestation

Estimates of the probability of infestation/infection, as influenced by seasonal and host variables, were modelled using generalised additive models (GAMs). We used a directed acyclic graph (DAG) to identify a set of models that reduce bias in the estimation of the total effect for each response variable (Fig. 2) following the approach of Arif and MacNeil [46]. The DAG structure represents hypothesised relationships within the system on the basis of bacterial, nycteribiid and host ecology, as well as study design, and it uses the presence or absence of nycteribiid parasitism, or bacterial infection on individual bats as the outcome (‘parasitism/infection’).

Microclimate factors (e.g. temperature, precipitation and humidity) are primary determinants of nycteribiid ecology and are expected to vary (1) geographically, (2) seasonally and (3) with interannual climate cycles [48–51]. Firstly, because these climatic factors demonstrate high concurvity when included together in a model, their individual effects are difficult to disentangle in a temporal or causal sequence. For example, temperature, humidity, precipitation and evaporation are correlated at a local scale and likely to demonstrate high concurvity if included in a GAM, resulting in unstable effect estimates for response variables. For this reason, ‘roost’ is included in the DAG as a geographic proxy variable for the complex of local climatic factors driving nycteribiid ecology. Secondly, the effect of the annual cycle on nycteribiid presence is assumed to operate entirely through seasonal changes in local climate, represented by an arrow connecting ‘season’ to ‘roost’ and using the month of sampling as the unit. Thirdly, interannual climate variation on the east coast of Australia occurs in cycles described by the ONI, with ONI anomaly (in °C) as units. This effect of interannual variation (‘ONI’) is assumed to influence the annual cycle (‘season’) as well as the local conditions (‘roost’), with both relationships represented with arrows in the DAG.

Host risk factors of interest for nycteribiid infestation and bacterial infection include host species, sex, age, reproductive status and body condition, each depicted in the DAG with a direct arrow to the outcome variable. Common body condition indices used in chiropteran species have been criticised as being no more effective at estimating fat stores than body mass [52]. We used a proxy measure of body condition (represented by ‘host condition’ in the DAG) determined by standardising body mass to a mean of zero and standard deviation of 1 within samples grouped by host species, sex and age.

The host variables expected to change throughout the year (age, reproductive status and body condition) are represented in the DAG with arrows from ‘season’ to ‘host age’, ‘host reproductive status’ and ‘host condition’ [30, 53, 54]. Australian flying foxes have an annual reproductive cycle, generally giving birth to a single pup once a year. Prior to weaning at ~5–6 months of age, pups are not sampled independently from their mother, meaning that our juvenile age category represents individuals of ~5–12 months of age and juveniles are not captured year-round [30]. The reproductive status of an individual is also dependent on both the age and sex. Juvenile bats are sexually immature and categorised as non-reproductive on the basis of our classification, and male and female bats have different reproductive biology, such that a male bat cannot be classed as pregnant or lactating. This is represented in the DAG as an arrow from each ‘host age’ and ‘host sex’ to ‘host reproductive status’. Body condition varies seasonally, with asynchronous cycles between male and female bats driven by differing energy requirements on the basis of reproduction and behaviour [54]. The dependency of body condition on an individual’s species, sex, age and the time of year is represented in the DAG by arrows from the corresponding variables to ‘host condition’. Finally, host species and sex composition within a roost may display temporally distinct dynamics, represented by arrows connecting ‘season’ to ‘host species’ and ‘host sex’.

We determined the appropriate co-variates for the model for each individual response variable using the backdoor criterion [47]. Briefly, the variable of interest (response variable), and any co-variate leading both directly to the response variable and via an alternative pathway to the outcome (‘parasitism/infection’), were identified as the minimum adjustment set required to estimate the probability of infestation/infection for the response variable. This was confirmed using the R package dagitty (v0.3–4) [55]. Only those variables identified as the minimum adjustment set required were included in the GAM estimating the effect for each response variable. GAMs were fitted using the mgcv (v1.9–1) package [56] with fast restricted maximum likelihood (fREML) smoothing parameter estimation. Concurvity, which represents the generalisation of the concept of collinearity to the GAM context, was assessed for each model using a cutoff of 0.6 on the estimated value of any variable pair applied. High concurvity values indicate increasing correlation between smooth terms conditional on the outcome. This has the effect of increasing the variance of model coefficients, in some cases resulting in unstable estimates. The outcome was nycteribiid presence on individual bats or blood-borne bacterial infection (i.e. 0 or 1), and shrinkage versions of thin plate smooths were used for numeric model covariates, while categorical variables were included as fixed effects, except for roost location, which was modelled using random intercepts. Season was always fitted as a cyclic cubic regression spline to allow January to follow December. No combination of variables resulted in a concurvity estimate > 0.6 once models, based on the minimum adjustment set for each response variable, were constructed. Exact formulae (in R syntax) for each model are described in Table S1 and summary output is described in Table S2. Prediction-based model inference was conducted using the R package marginaleffects (v0.25.1) [57].

#### Comparison of environmental variables among roost locations

We performed an ordination via a principal components analysis (PCA) to explore the variation of local climatic factors across sampling sessions using prcomp() from R [41]. The abiotic data used for each sampling session included temperature, solar radiation, precipitation, evaporation and humidity, all of which were centred and scaled prior to performing the PCA. We then conducted a permutational multivariate analysis of variance (PERMANOVA) to statistically compare the effect of roost location on the resultant principal component scores using a cutoff of *P* = 0.05 in vegan [58] and visualised the ordination using the package ggbiplot (v0.6.2) [59].

#### Estimating probability of blood-borne bacterial infection given nycteribiid parasitism

We used logistic regression with glm() in base R to estimate the risk of bacterial infection on the basis of nycteribiid parasitism. Bacterial infection was the outcome variable and nycteribiid presence (binary) was the predictor variable. A separate model was run for each *Bartonella* spp. and *Borrelia* spp.

## Results

### *Cyclopodia albertisii* is the predominant ectoparasite on black and grey-headed flying foxes

Nycteribiid parasites (*n* = 603) were collected from individual bats (*n* = 529) during 16 sessions from February 2018 till January 2019. The mean number of bat flies collected per session was 39.4 (range: 5–85), and the mean collected per individual was 1.1 (range: 1–5). Both female (*n* = 207) and male (*n* = 423) parasites were collected, and gravid females were observed throughout the sampling period. The proportion of female flies per session sample ranged from 0.11 to 0.5, reaching minimum values in July–August (Supplementary Fig. S1).

**Fig. 3:**
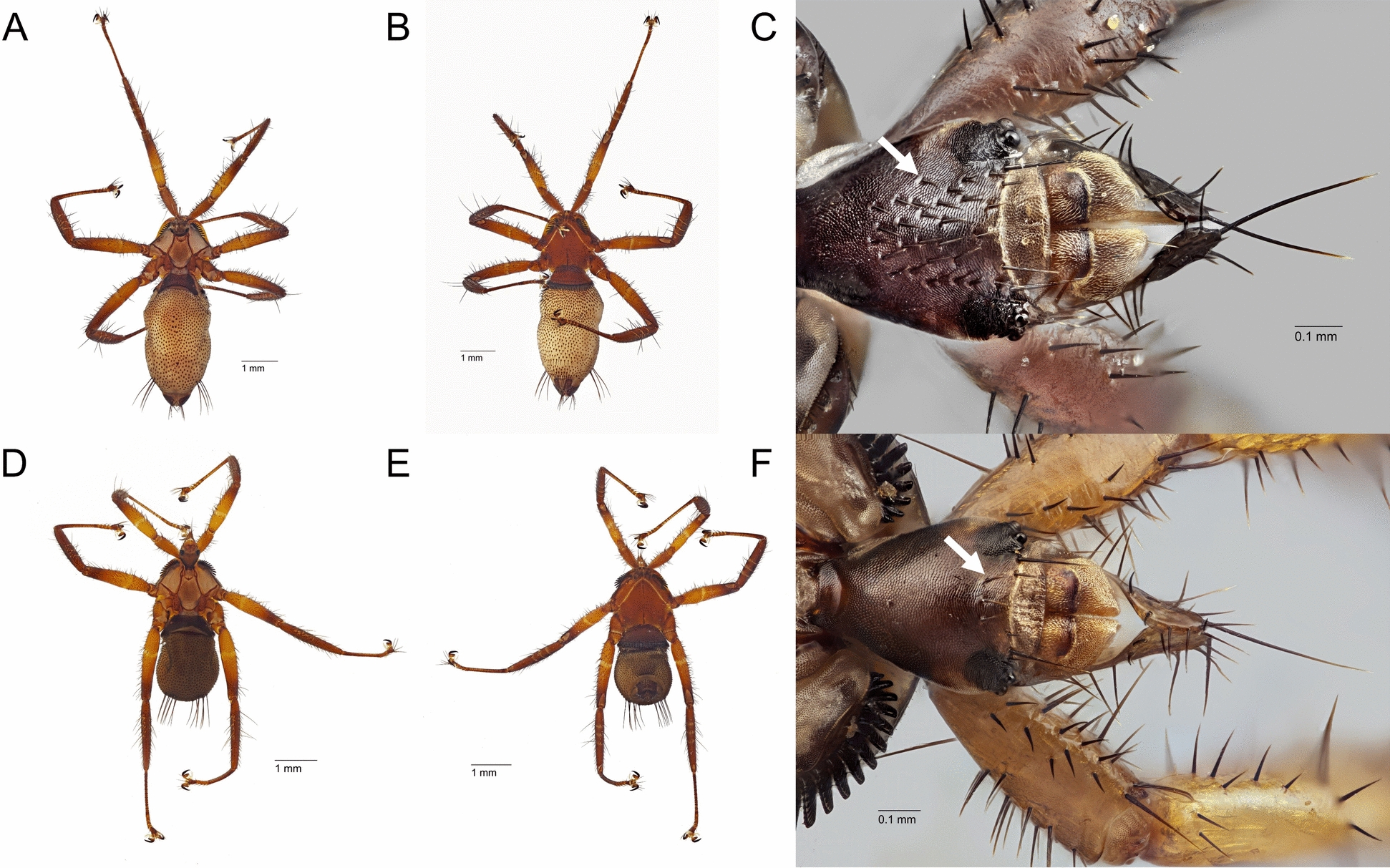
Images of nycteribiid bat flies of the genus *Cyclopodia* collected from flying foxes during the study. Note the difference in number of setae on the dorsum of the head of *C. albertisii* (**C**, many setae indicated by the arrow) compared with *C. australis* (**F**, few setae indicated by the arrow). **A**, **B** Female *C. albertisii*, ID Code ACGMP001-36, Gympie, 31/1/2019 from host bat *Pteropus poliocephalus*, (**A**) dorsal habitus, (**B**) ventral habitus. **C** Dorsal view of the head of a male *C. albertisii*, ID Code ACRED004-56, Redcliffe, 14/12/2018 from host bat, *P. alecto*. **D**, **E** Female *C. australis*, ID Code ACMOM001-10, Mount Ommaney, 17/1/2019 from host bat *P. poliocephalus*, (**D**) dorsal habitus, (**E**) ventral habitus. **F** Dorsal view of the head of a female *C. australis*, ID Code ACMOM001-41, Mount Ommaney, 17/1/2019 from host bat *P. alecto*. All images by Lily Kumpe, Queensland Museum, taken using a Visionary Digital BK imaging system and a Canon 5DS camera with either a Canon 200 mm lens and 20× Mitutoyo microscope objective (**A**, **B**, **D**, **E**) or a Canon 65 mm MP-E lens at 2.5× (**C**, **F**), and with multiple source images focus-stacked with Zerene stacker software [60]

Most specimens were identified as *Cyclopodia albertisii* (Rondani 1878; *n* = 593); however, a small number of *C. australis* (Theodor 1959; *n* = 10) specimens were collected from both black flying foxes and grey-headed flying foxes in Toowoomba, Mount Ommaney, Gympie and Redcliffe (Fig. 3). Both *Cyclopodia* species were found co-parasitising a single black flying fox in Redcliffe.

### Nycteribiid parasitism displays seasonal dynamics, with peak prevalence and median intensity in autumn

Nycteribiid data were collected over 42 sessions across nine roost locations between August 2017 and September 2022, with presence recorded for 2235 individual bats and intensity recorded for 2168 individuals (Table 1). Sampling sessions occurred primarily at two roost locations—Redcliffe (*n* = 17 sessions) and Toowoomba (*n* = 16 sessions)—with the remaining seven locations having only 1–2 sessions each. Black flying foxes were the primary target species (*n* = 2018 individuals sampled) and were captured at all locations except Maclean. Grey-headed flying foxes (*n* = 217 individuals sampled) were captured at all locations except Bundaberg and Clunes. Some bats caught still had dependent pups attached (*n* = 37 bats), which were not examined separately for ectoparasites and were excluded from further analysis.

**Table 1:** Summary table of study sampling effort by site and host variables

Site	No. of sessions	No. individuals caught	Prevalence (95% CI)	Individuals intensity recorded	BFF	GHFF	Adults	Subadults	Juveniles	Females	Males
Bundaberg	1	17	100.0 (80.5–100.0)	17	17	0	11	3	3	9	8
Clunes	2	51	51.0 (36.6–65.2)	51	51	0	43	2	6	25	26
Gympie	1	62	95.2 (86.5–99.0)	59	59	3	31	2	29	32	30
Hervey Bay	2	130	73.1 (64.6–80.5)	130	66	64	81	28	21	64	66
Maclean	1	58	44.8 (31.7–58.5)	58	0	58	31	5	22	29	28
Mount Ommaney	1	60	90.0 (79.5–96.2)	60	29	31	45	7	8	27	33
Redcliffe	17	979	78.4 (75.7–81.0)	977	945	34	709	96	174	441	538
Sunnybank	1	62	96.8 (88.8–99.6)	0	45	17	39	16	7	25	37
Toowoomba	16	816	46.4 (43.0–49.9)	816	806	10	603	79	134	423	393
Total	42	2235	66.4 (64.4–68.3)	2168	2018	217	1593	238	404	1075	1159

Dependent-pups that were caught still attached to their mothers (*n* = 37) were not examined for ectoparasites and are excluded from the table and further analysis

BFF, black flying foxes (*Pteropus alecto*); GHFF, grey-headed flying foxes (*P. poliocephalus*)

**Fig. 4:**
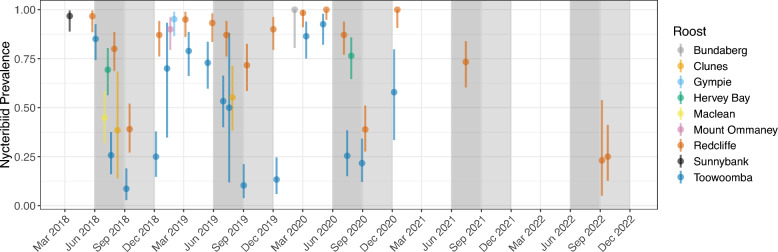
Nycteribiid prevalence and 95% binomial confidence intervals coloured by roost location. Each observation represents a separate catching session. Dark grey shading from 1 June till 31 August indicates the winter period. Light-grey shading from 1 September till 30 November indicates spring

**Fig. 5:**
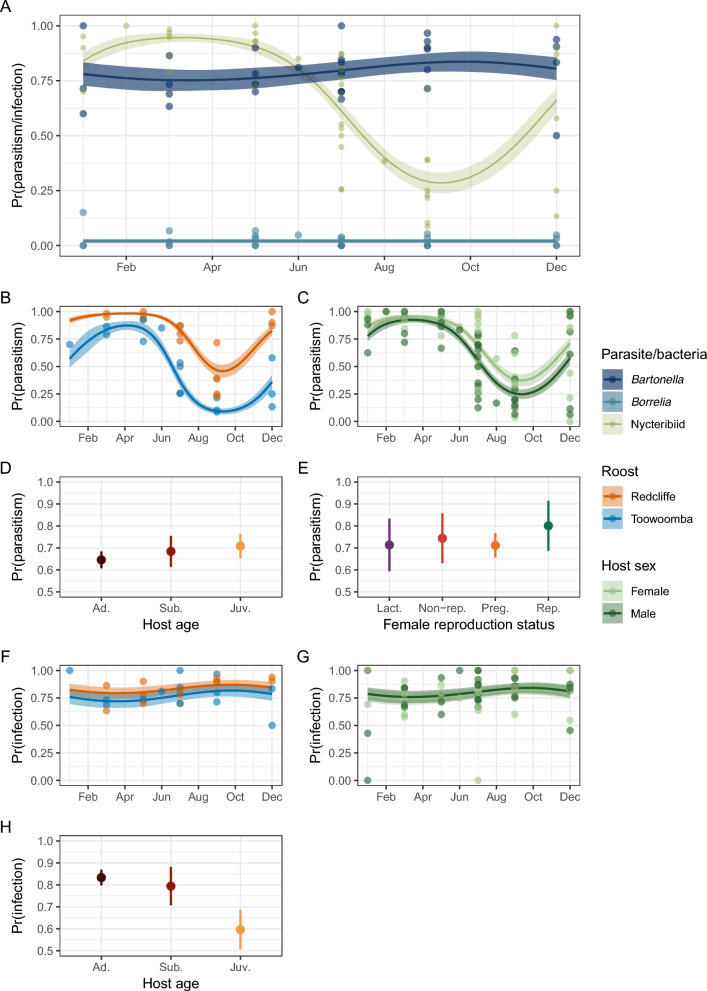
Model-based conditional predictions and 95% confidence intervals of probability of nycteribiid parasitism and blood-borne bacterial infection. **A** Seasonal probability of parasitism/infection with nycteribiids, *Bartonella* spp. and *Borrelia* spp. Nycteribiid prediction is conditional on a fixed ONI mean of 0.14, while *Bartonella* spp. and *Borrelia* spp. prediction uses a fixed ONI mean of 0.28. Points are the prevalence for each session plotted by the month sampling was undertaken. **B** Seasonal probability of nycteribiid parasitism at Redcliffe and Toowoomba roosts. Points are the prevalence for each session plotted by the month sampling was undertaken. **C** Probability of nycteribiid parasitism by host sex. Points are host sex-specific prevalence for each session plotted by month. **D** Probability of nycteribiid parasitism by host age category (Ad., adult; Sub., subadult; Juv., juvenile) conditional on a mean month of 6.83 (25 June). **E** Probability of nycteribiid parasitism by reproductive status in female bats (Lact., lactating; Non-rep., non-reproductive; Preg., pregnant; Rep., reproductive) conditional on a mean month of 6.77 (23 June) and age fixed as adult. Not all host reproduction stages are compatible with this time of year; however, this adjusts for the non-linear effect of season on risk of parasitism and facilitates comparison between the stages. **F** Seasonal probability of *Bartonella* spp. infection at Redcliffe and Toowoomba roosts. Points are the prevalence for each session plotted by the month sampling was undertaken. **G** Probability of *Bartonella* spp. infection by host sex. Points are host sex-specific prevalence for each session plotted by month. **H** Probability of *Bartonella* spp. infection by host age category (Ad., adult; Sub., subadult; Juv., juvenile) conditional on a mean month of 6.53 (16 June)

Nycteribiids were ubiquitous, being detected at every session and roost location. The lowest recorded session prevalence was 8.6% (95% CI 2.9–19.0%) during September 2018 in Toowoomba, while 100% prevalence was observed on three occasions: twice in Redcliffe during May 2020 (95% CI 94.8–100.0%) and December 2020 (95% CI 90.7–100.0%), and once in Bundaberg in February 2020 (95% CI 80.5–100.0%) (Fig. 4). Our analysis identified seasonal trends in the probability of nycteribiid parasitism on individuals (Fig. 5A), and the prevalence and session median intensity of bat flies per host. Parasitism increased from December through summer to peak in autumn (March–May), followed by a decrease in winter (July) to the nadir at the beginning of spring (September) (Fig. 4; Supplementary Fig. S2). Some interannual variation was observed, with the spring session at Redcliffe in 2019 having a high prevalence relative to the previous and subsequent years. There was poor support for this interannual effect being due to the 3-month average ONI in our ‘ONI’ model estimate; however, our model did not account for potential lags in the effect of ONI (Supplementary Fig. S3). The observed dynamics in prevalence and median intensity were accompanied by inverse changes in parasite distribution within the roost (Supplementary Fig. S4). That is, as nycteribiid prevalence and median intensity decreased, aggregation increased, and fewer host individuals harboured most parasites.

### Local abiotic factors differ significantly between the longitudinally sampled roosts

Nycteribiid prevalence and session median intensity were consistently higher at Redcliffe compared with Toowoomba (Fig. 4; Supplementary Fig. S2), with the most pronounced differences during the winter to spring period (Fig. 5B). The PCA of abiotic factors demonstrated that these locations differed primarily in humidity levels, with sessions clustering by roost location along the humidity-dominated PC2 axis (Supplementary Fig. S5; Supplementary Table S3), as confirmed through PERMANOVA (*F*_8,32_ = 4.16, *R*^2^ = 0.51, *P* = 0.004). Overall, session prevalence was highest in conditions combining high humidity (at the positive extremes of PC2) with high temperature and low evaporation (negative extremes of PC1).

### Nycteribiid parasitism differs on the basis of host sex

Host sex was the only host-related variable that clearly affected the probability of nycteribiid parasitism in our analysis, with female bats having higher parasitism than males (Fig. 5C). The difference fluctuated seasonally, being greatest from winter till early summer (June–December) coinciding with the mid gestational period through till early lactation in the female bats’ reproductive cycle. Our model suggested a slight decrease in the probability of parasitism with age (from juveniles, to subadults, then adults); however, this difference was not deemed significant on the basis of overlapping confidence intervals of conditional predictions (Fig. 5D). Similarly, predictions for female reproductive status (Fig. 5E), male reproductive status (Supplementary Fig. S6), and host species (Supplementary Fig. S7) did not differ significantly. The effect of body condition (standardised body mass) on the probability of nycteribiid parasitism was also non-significant but suggested a slightly positive linear relationship, the slope of which did not change seasonally (Supplementary Fig. S8).

### *Bartonella* spp. demonstrate asynchronous seasonality to nycteribiids while *Borrelia* spp. were rarely detected

*Bartonella* and *Borrelia* PCR screening was undertaken on blood samples from 840 bats (837 black flying foxes and three grey-headed flying foxes) collected across 33 sessions at six roosts between May 2018 and September 2020. Two monophyletic clades of *Bartonella* consisting of 55 distinct *gltA* sequence variants (previously reported in [32]) and two haplotypes of *Borrelia* (previously reported in [15]) were identified (Supplementary Fig. S9). *Bartonella* spp. infection was pervasive, with a total of 664 bats testing positive (78.8%, 95% CI 75.9–81.5%), and were detected at all sessions except for Maclean in July 2018, where only a single grey-headed flying fox had a sample submitted for screening. Otherwise, the lowest prevalence recorded was 50% (95% CI 28.2–71.8%, at Toowoomba in December 2018). Twice, the prevalence was 100% (at Toowoomba in January 2019 [*n* = 9, 95% CI 66.4–100.0] and July 2019 [*n* = 6, 95% CI 54.1–100.0]); however, few bats were captured. The next highest recorded prevalence was 96.7% (95% CI 82.8–99.9%) in Toowoomba in September 2019, where 30 bats were screened. Our model weakly supported asynchronous cycles of *Bartonella* spp. prevalence in comparison with nycteribiids, increasing through winter to a peak in spring and decreasing over summer within a smaller range than displayed by nycteribiids (Fig. 5A). Unlike nycteribiids, the probability of *Bartonella* spp. infection did not differ significantly by roost location or host sex (Fig. 5F and G) but did differ by host age, with juvenile bats displaying a lower probability of infection (Fig. 5H) (previously reported in [32]). Logistic regression with nycteribiid infestation as the predictor did not support a significant association between host *Bartonella* spp. infection and nycteribiid parasitism (OR 1.09, 95% CI 0.76–1.55, *Z* = 0.47, *P* = 0.64).

Most individual bats that were positive for *Borrelia* spp. were co-infected with *Bartonella* spp. (16/17, 94.1%). Host metadata for individual bats positive for *Borrelia* spp. is reported in Verrett et al. [15]. *Borrelia* spp. were detected in 13 sessions with a peak prevalence of 15.0% (95% CI 3.2–37.9%) at Gympie in January 2019, and the lowest recorded prevalence of 1.7% (95% CI 0.0–9.2%) in Toowoomba in March 2019. There were insufficient detections to infer potential seasonality in prevalence, and this is reflected in the model estimate (which is a constant 0% throughout the year; Fig. 5A). Further analysis of potential host risk factors for *Borrelia* spp. was not performed owing to the low number of detections. Logistic regression was equivocal whether *Borrelia* spp. infection was associated with nycteribiid parasitism (OR 7.09, 95% CI 1.44–128.35, *Z* = 1.89, *P* = 0.06), likely because the small number of positive individuals limited the power of this analysis.

## Discussion

To better understand ectoparasite and pathogen ecology in Australian flying foxes, we conducted a longitudinal investigation of their ectoparasite dynamics, examining seasonal and host-related factors driving temporal and spatial patterns in parasitism. We surveyed two species of flying foxes at multiple roost locations across an approximate 3-year period for the presence and intensity of ectoparasites. In addition, blood samples collected from a subset of individuals were screened for blood-borne bacteria (*Bartonella* and *Borrelia* spp.). We found that nycteribiid bat flies were predominantly *C. albertisii* and displayed a distinct seasonality in their prevalence and intensity, peaking in autumn before waning through winter and spring. The magnitude of both prevalence and intensity varied spatially, likely owing to local abiotic factors such as humidity. Infection with the bacteria *Bartonella* spp. was consistently high throughout the study period, though did demonstrate moderate seasonality that appeared to be asynchronous with nycteribiid prevalence, while *Borrelia* spp. detections were rare. The probability of nycteribiid parasitism differed by host sex, with females having an increased probability compared with males, while the probability of *Bartonella* spp. infection differed by host age, with juveniles having a decreased probability compared with subadults and adults (as reported in [32]). By documenting these associations, this study informs broader understanding of how parasite ecology and potential haematophagous vector-driven pathogen transmission within highly mobile, gregarious host communities may contribute to zoonotic spillover.

We observed distinct seasonal fluctuations in both the prevalence and intensity of nycteribiid bat flies. This phenomenon is consistent with many other bat fly systems involving a variety of host and parasite species [24, 35, 61, 62]. A similar longitudinal study of nycteribiid (*Eucampsipoda madagascarensis*) parasitism of pteropodids (*Rousettus madagascariensis*) in central Madagascar reported almost identical seasonal dynamics, with a single annual peak in bat fly intensity towards the end of the rainy season in March [63]. The authors also report higher nycteribiid abundance on female bats [63]. We similarly report a significantly higher risk of nycteribiid parasitism for female bats. Both male and female host bias has been observed in different nycteribiid systems with sex-based differences in host behaviour, physiology and parasite preference cited as explanations [35, 61, 64]. We failed to detect a difference in parasitism by reproductive status in female bats that would support reproductive physiology as the driver for sex-based difference in parasitism; however, further investigation is required to determine the underlying cause, and the impact it may have on potential parasite-mediated transmission.

Our study identifies poor support for an effect of host body condition on nycteribiid parasitism risk in Australian flying foxes. This contrasts the above Malagasy study, where an effect was identified for both study species, *R. madagascariensis* and *Eidolon dupreanum* [63]. A positive relationship between parasite load and body condition (measured by body mass: forearm residual) was identified for the smaller *R. madagascariensis* (~60 g) while the reverse was true for the larger *E. dupreanum* (~250 g). The authors suggested the increasing surface area of the smaller species compared with a potential immunocompromise from nutritional deficit in the larger species as potential mechanisms [63]. The disparity with our study findings could be due to the lack of sensitivity in using a binary measure of parasitism rather than intensity, and that parasitism is more strongly shaped by variables other than body condition in the much larger black flying fox and grey-headed flying fox (~400–1000 g) or due to differences in the indices used (we have standardised by host species, sex and age class while the Malagasy study did not). This warrants further investigation in the role of body condition in shaping parasite-mediated transmission during periods of dietary resource restriction for the host.

We hypothesise that nycteribiid parasitism in Australian flying foxes is primarily driven by local climatic factors rather than by host reproduction. Roost microclimate has previously been shown to influence bat fly parasitism [26, 50]. Another study reporting nycteribiid prevalence in Australian flying foxes found reduced prevalence in roosts from the southern latitudes [26]. Our work expands on this by longitudinally measuring prevalence at two sites with contrasting local climates. Toowoomba is an inland plateau, approximately 120 km from the coast and with 691 m of elevation, while Redcliffe is a low-lying, coastally located roost. We identified differences in abiotic gradients—namely humidity, temperature and evaporation—between roost sites, which provides evidence for the possible role of local climatic factors in driving nycteribiid parasitism. The strict osmoregulatory requirements of terrestrial arthropods make climatic variables such as humidity and precipitation crucial for determining arthropod distribution and seasonal occurrence [65], while temperature also impacts insect physiology and behaviour [66]. Both the osmoregulatory requirements and temperature dependent physiology and behaviour of arthropods provides a parsimonious explanation for the mechanisms through which climatic variables could impact seasonal and spatial occurrence of nycteribiid parasitism. The role of climatic drivers is further supported by findings in Andrianiaina et al. [63], where temperature and precipitation were also identified as important predictors of nycteribiid seasonality. Environmental conditions have been linked to nycteribiid reproduction and survival, with reduced winter fertility in temperate species compared with equatorial species [35, 67] and compromised pupal development in species parasitising hosts that favour exposed roosts (canopy) compared with caves [68]. Here, gravid female nycteribiids were identified throughout the sampling period, suggesting the seasonal dynamics may instead be a result of reduced pupal development in the colder, drier winter period when prevalence decreases. More complete sampling of nycteribiids to quantify seasonal trends in fecundity, as well as experimental investigation of the impact of abiotic factors on pupal development success, and nycteribiid reproductive activity is required to improve understanding of the process through which climatic factors drive nycteribiid parasitism in Australian flying foxes.

The asynchronous seasonal pattern between nycteribiid parasitism and detection of *Bartonella* DNA in our study contrasts with findings from a similar system in *Pteropus medius* (Pteropodidae) in Bangladesh [69]. That study reported an increase in *Bartonella* spp. prevalence in bats coinciding with an increase in nycteribiid prevalence, citing it as support of nycteribiid-mediated transmission. The authors detected *Bartonella* DNA in both bats and bat flies and demonstrated identical phylogenetic clades of *Bartonella* present in both bats and bat flies, providing strong support for transmission between the host and parasites [69]. Our study has a larger sample size collected over a longer duration and across multiple roosts, improving our ability to compare trends in the occurrence of both nycteribiids and *Bartonella* spp.; however, we did not screen bat flies for *Bartonella* DNA and thus cannot explore phylogenetic evidence for transmission. Long-lasting intra-erythrocytic bacteremia in mammalian hosts is a feature of *Bartonella* infection and likely accounts for the consistently elevated prevalence that we observed [70]. The strong effect of host age on the probability of *Bartonella* spp. infection suggests that transmission via nycteribiid vectors occurs commonly; however, the persistent bacteremia obscures potential evidence of seasonal variation in vector-borne transmission when relying on prevalence alone. Further investigation into the infection dynamics of *Bartonella* in Australian flying foxes is recommended to better characterise its utility as a model organism for exploring vector transmission arising from nycteribiids and the potential zoonotic spillover risk posed by the persistently high prevalence in flying foxes reported here.

While we progressed knowledge of Australian flying fox nycteribiid ecology, there are limitations to our study. Parasite counts on individuals were right truncated with a 10+ category, limiting analysis of variables driving the intensity of parasitism. This may have reduced the sensitivity of model estimates for host factors such as body condition, which may have benefited from a quantitative estimate rather than binary probability of parasitism. We were unable to explore the impact host population structure within roosts may have had on the seasonal prevalence of nycteribiid and blood-borne bacteria. This may have contributed to differences in nycteribiid prevalence observed between roosts. Another limitation stems from the abiotic variables used to explore local climatic differences between roost sites, which were collected from publicly available remote sensing data, and thus may not accurately reflect conditions in the roost canopy. Use of abiotic data collected directly from within the roosts may improve future analysis of the influence of abiotic factors on nycteribiids. In addition, we found limited support for an interannual effect of climate driven by the ONI on nycteribiid parasitism; however, this may require time periods longer than this study permitted to establish any relationship. As a point of comparison, the link between spillover of HeV from Australian flying foxes and food shortages following strong El Niño events (ONI > 0.8) was demonstrated in a study using 25 years of data [71]. Longer study periods and inclusion of temporally lagged parameters in model analysis could improve the ability to detect interannual effects on parasitism and blood-borne pathogen prevalence, and potential interaction with established ecological drivers of spillover. Increased study duration would facilitate a greater number of observations and lagged parameters would allow investigation of temporal dependencies that may be important for parasite development or other aspects of host and pathogen ecology. Finally, identifying nycteribiids as vectors of *Bartonella* spp. in flying foxes cannot be determined solely by comparing spatial or temporal patterns in prevalence. This would require infection in nycteribiids, ideally coupled with temporal and spatial patterns in *Bartonella* genetic similarity in both nycteribiids and flying foxes and direct transmission experiments.

## Conclusions

We detail here aspects of nycteribiid ecology critical for understanding their vector potential within flying fox roosts while also providing direction for future research both in nycteribiid and blood-borne bacterial ecology in Australian flying foxes. Nycteribiid parasitism on black flying foxes (*P. alecto*) and grey-headed flying foxes (*P. poliocephalus*) in eastern Australia fluctuates in a consistent annual cycle, peaking in late summer and dropping off during the winter period. This appears to be dependent on local abiotic factors operating at the roost level. Differences in parasitism between host sex were apparent, though understanding the mechanism underpinning this variation will require further investigation. Nycteribiid and *Bartonella* spp. prevalence displayed asynchronous peaks, indicating that further research into the epidemiology of both is required to better understand nycteribiid-mediated transmission dynamics.

## Supplementary Information

Supplementary Material 1.

Supplementary Material 2.

Supplementary Material 3.

Supplementary Material 4.

Supplementary Material 5.

Supplementary Material 6.

Supplementary Material 7.

Supplementary Material 8.

Supplementary Material 9.

Supplementary Material 10.

## Data Availability

The datasets generated and/or analysed during the current study are available in the Sydney eScholarship repository, 10.25910/gsyn-4294.
