# Supplementary figures and images for: Asynchronous seasonal dynamics of nycteribiid bat flies and Bartonella spp. in Australian flying foxes (Pteropus spp.)
Source: Parasit Vectors. 2026 Jan 29;19:96. doi: 10.1186/s13071-026-07243-1 (PMC12924405; doi:10.1186/s13071-026-07243-1)

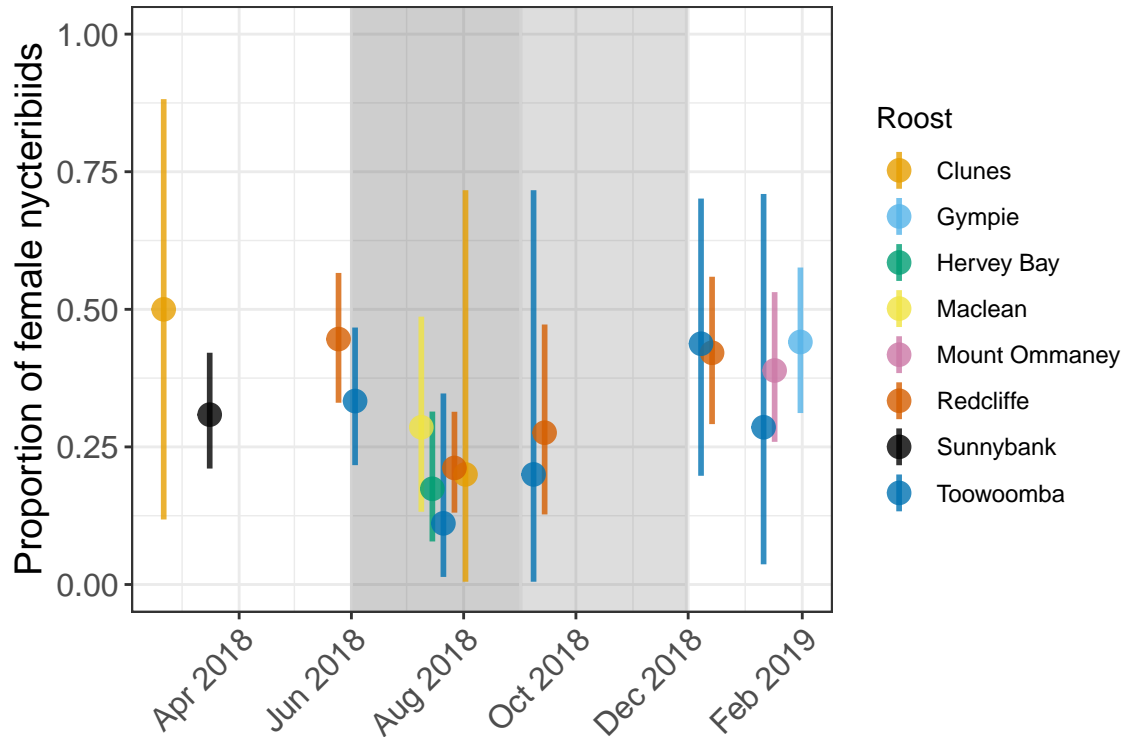

Supplement: Supplementary file 1 — Supplementary Material 1. [file 13071_2026_7243_MOESM1_ESM.pdf]

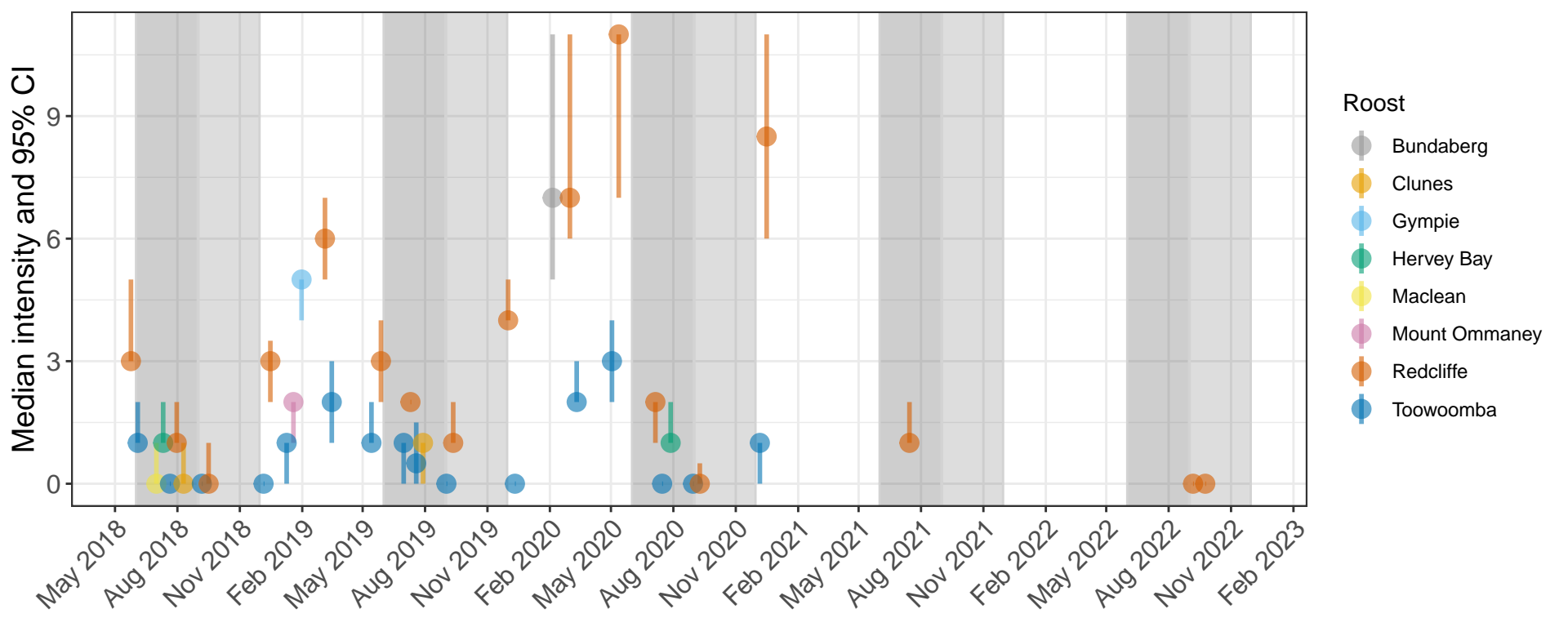

Supplement: Supplementary file 2 — Supplementary Material 2. [file 13071_2026_7243_MOESM2_ESM.pdf]

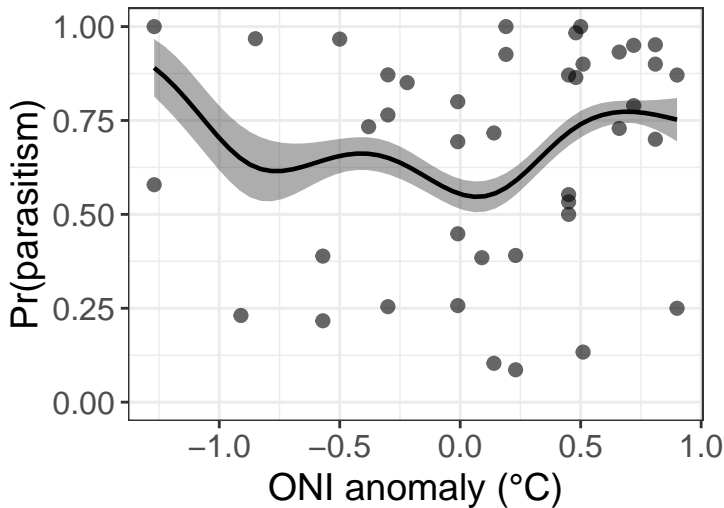

Supplement: Supplementary file 3 — Supplementary Material 3. [file 13071_2026_7243_MOESM3_ESM.pdf]

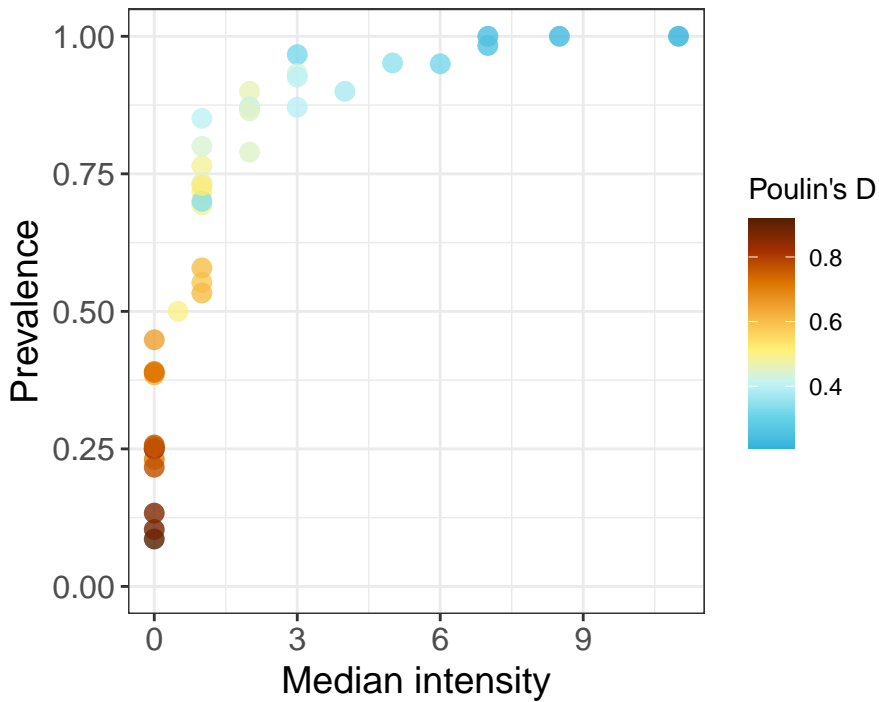

Supplement: Supplementary file 4 — Supplementary Material 4. [file 13071_2026_7243_MOESM4_ESM.pdf]

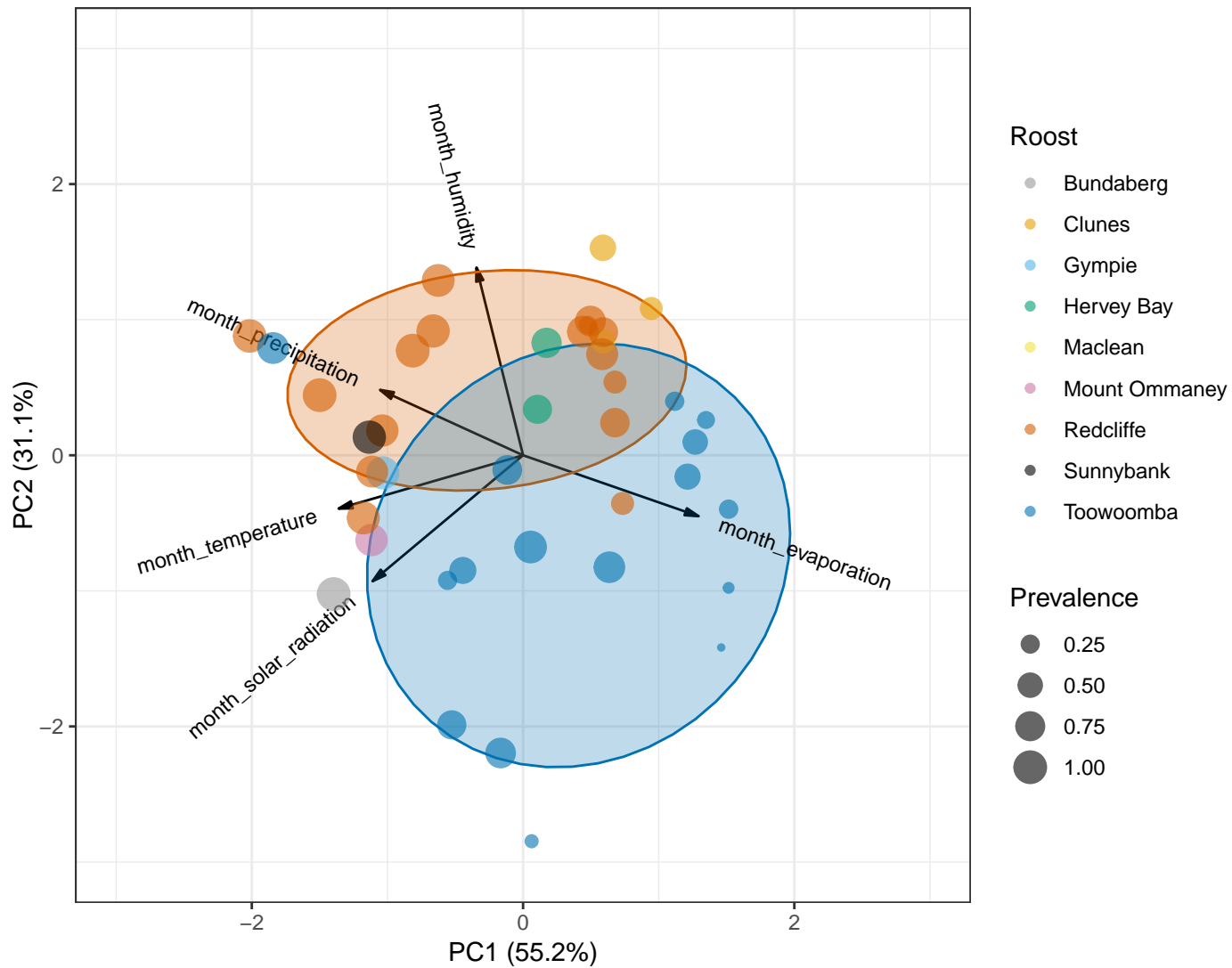

Supplement: Supplementary file 5 — Supplementary Material 5. [file 13071_2026_7243_MOESM5_ESM.pdf]

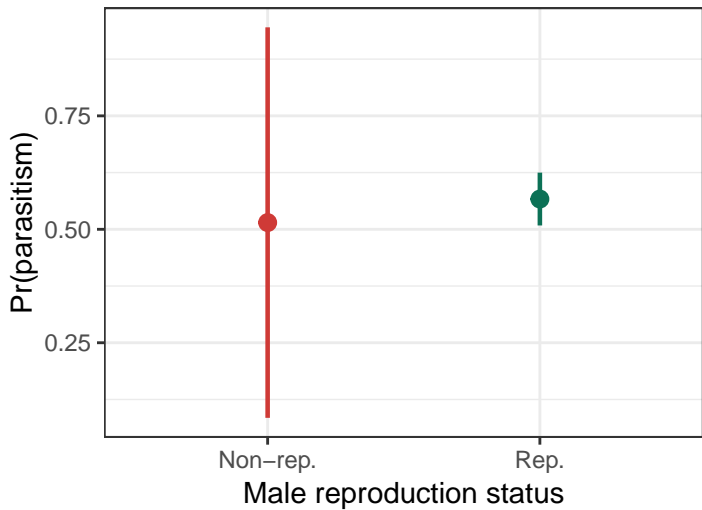

Supplement: Supplementary file 6 — Supplementary Material 6. [file 13071_2026_7243_MOESM6_ESM.pdf]

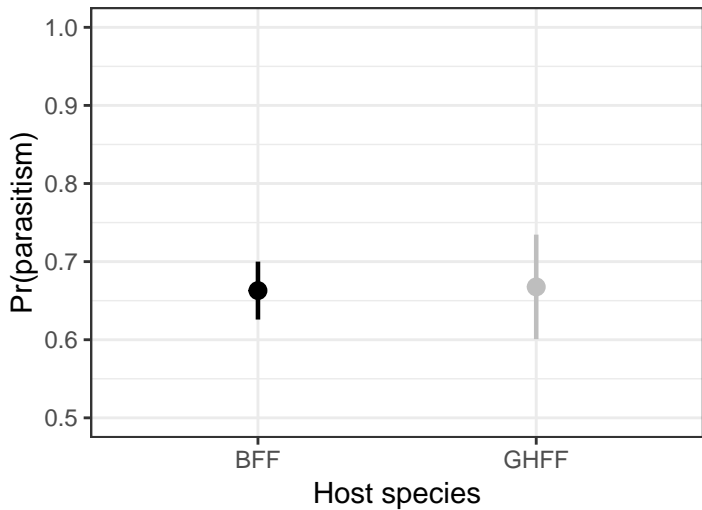

Supplement: Supplementary file 7 — Supplementary Material 7. [file 13071_2026_7243_MOESM7_ESM.pdf]

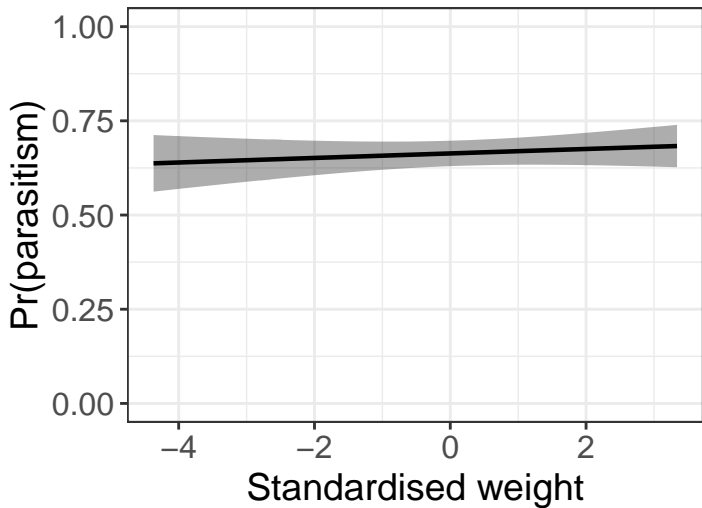

Supplement: Supplementary file 8 — Supplementary Material 8. [file 13071_2026_7243_MOESM8_ESM.pdf]

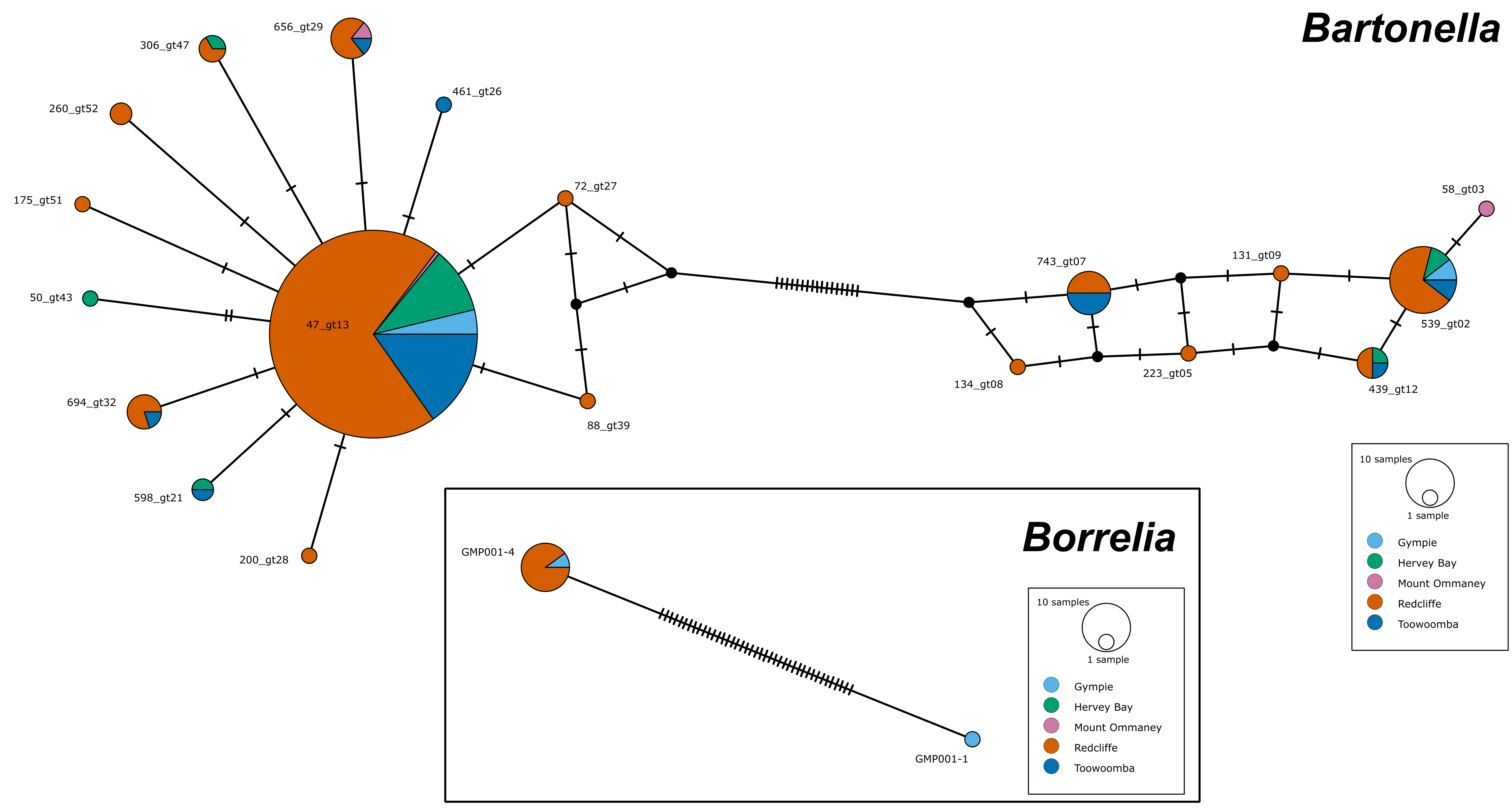

Supplement: Supplementary file 9 — Supplementary Material 9. [file 13071_2026_7243_MOESM9_ESM.png]
