# Supplementary material for: Asynchronous seasonal dynamics of nycteribiid bat flies and Bartonella spp. in Australian flying foxes (Pteropus spp.)
Source: Parasit Vectors. 2026 Jan 29;19:96. doi: 10.1186/s13071-026-07243-1 (PMC12924405; doi:10.1186/s13071-026-07243-1)
Supplement: Supplementary file 10 — Supplementary Material 10. [file 13071_2026_7243_MOESM10_ESM.docx]

# Supplementary Information

Table S1: Model structure for estimates of the probability of nycteribiid infestation and bacterial infection in flying foxes within the study.

| Model | Variable of interest | Outcome (y) | Minimum adjustment set | Model formulae | Comments |
| --- | --- | --- | --- | --- | --- |
| Season | Season | Parasitism/ Infection | ONI | y ~ s(season, bs = “cc”, k = 6) + s(ONI, bs = “ts”, k = 6) |  |
| ONI | ONI | Parasitism/ Infection | - | y ~ s(ONI, bs = “ts”, k = 6) |  |
| Roost | Roost | Parasitism/ Infection | Season | y ~ s(season, bs = “cc”, k = 6) + s(roost, bs = “re”) |  |
| Host age | Host age | Parasitism/ Infection | Season | y ~ s(season, bs = “cc”, k = 6) + age |  |
| Host sex | Host sex | Parasitism/ Infection | Season | y ~ s(season, bs = “cc”, k = 6) + sex |  |
| Host reproductive status | Host reproductive status | Parasitism/ Infection | Season, age, sex | y ~ s(season, bs = “cc”, = 6) + age  + reproductive_status | Data was split by sex and separate models run for each. |
| Host species | Host species | Parasitism/ Infection | Season | y ~ s(season, bs = “cc”, k = 6) + species |  |
| Host condition | Host condition | Parasitism/ Infection | Season, species, sex, age | y ~ s(season, bs = “cc”, k = 6) + s(condition, bs = “ts”) |  |

The minimum adjustment set includes the variables of interest and any additional variables required to reduce bias in the estimate based on our assumptions of the host-parasite system. This was determined by applying the backdoor criteria to the DAG in Fig. 2 of the main text. Generally, each variable of the minimum adjustment set was included as a term in the model formula, however in circumstances where this resulted in excessive model concurvity (>0.6), variables were controlled for by stratification or unit standardisation (in the case of host condition). This is outlined in the comments for each model.

Table S2: Summary model output for each GAM and logistic regression model.

| Model | Parametric coefficients | | | | | Smooth terms | | | | | R^2^-adj | Dev. | fREML |
| --- | --- | --- | --- | --- | --- | --- | --- | --- | --- | --- | --- | --- | --- |
|  | Coefficient | Est. | SE | Z | p | Term | EDF | RDF | Χ^2^ | p |  |  |  |
| *Nycteribiid GAM* |  |  |  |  |  |  |  |  |  |  |  |  |  |
| Season | (Intercept) | 1.38 | 0.09 | 14.77 | <0.001* | Season | 3.73 | 4 | 337.2 | <0.001* | 0.202 | 17.2 | 3180.1 |
|  |  |  |  |  |  | ONI | 0.99 | 5 | 13.4 | <0.001* |  |  |  |
| ONI | (Intercept) | 0.78 | 0.05 | 15.17 | <0.001* | ONI | 4.67 | 5 | 75.8 | <0.001* | 0.036 | 3.1 | 3380 |
| Roost | (Intercept) | 1.46 | 0.34 | 4.35 | <0.001* | Season | 3.75 | 4 | 10611.8 | <0.001* | 0.344 | 29.2 | 3087.1 |
|  |  |  |  |  |  | Roost | 6.44 | 9 | 280.1 | <0.001* |  |  |  |
| Host age | (Intercept) | 1.06 | 0.07 | 14.19 | <0.001* | Season | 3.70 | 4 | 355.5 | <0.001* | 0.207 | 17.4 | 3245 |
|  | Juvenile | 0.29 | 0.14 | 2.05 | 0.04* |  |  |  |  |  |  |  |  |
|  | Subadult | 0.17 | 0.17 | 1.02 | 0.31 |  |  |  |  |  |  |  |  |
| Host sex | (Intercept) | 1.45 | 0.09 | 16.11 | <0.001* | Season | 3.71 | 4 | 357.9 | <0.001* | 0.220 | 18.5 | 3228.3 |
|  | Male | -0.59 | 0.10 | -5.80 | <0.001* |  |  |  |  |  |  |  |  |
| Host reproductive status (female) | (Intercept) | 1.31 | 0.23 | 5.70 | <0.001* | Season | 3.36 | 4 | 119.1 | <0.001* | 0.177 | 16.2 | 1525.6 |
|  | Non-repro | 0.15 | 0.39 | 0.39 | 0.69 |  |  |  |  |  |  |  |  |
|  | Pregnant | -0.01 | 0.31 | -0.31 | 0.97 |  |  |  |  |  |  |  |  |
|  | Repro | 0.49 | 0.43 | 1.11 | 0.26 |  |  |  |  |  |  |  |  |
|  | Juvenile | 0.07 | 0.34 | 0.22 | 0.82 |  |  |  |  |  |  |  |  |
|  | Subadult | 0.13 | 0.35 | 0.38 | 0.71 |  |  |  |  |  |  |  |  |
| Host reproductive status (male) | (Intercept) | 0.60 | 0.88 | 0.69 | 0.49 | Season | 3.56 | 4 | 201.8 | <0.001* | 0.224 | 18.6 | 1683.3 |
|  | Repro | 0.21 | 0.88 | 0.24 | 0.81 |  |  |  |  |  |  |  |  |
|  | Juvenile | 0.50 | 0.89 | 0.56 | 0.57 |  |  |  |  |  |  |  |  |
|  | Subadult | 0.27 | 0.88 | 0.30 | 0.76 |  |  |  |  |  |  |  |  |
| Host species | (Intercept) | 1.12 | 0.07 | 16.15 | <0.001* | Season | 3.70 | 4 | 357.0 | <0.001* | 0.205 | 17.2 | 3246.3 |
|  | GHFF | 0.02 | 0.17 | 0.13 | 0.9 |  |  |  |  |  |  |  |  |
| Host condition | (Intercept) | 0.98 | 0.06 | 15.88 | <0.001* | Season | 3.71 | 4 | 359.9 | <0.001* | 0.207 | 17.4 | 3231.3 |
|  |  |  |  |  |  | Host condition | 0.40 | 9 | 0.7 | 0.19 |  |  |  |
| *Bartonella GAM* |  |  |  |  |  |  |  |  |  |  |  |  |  |
| Season | (Intercept) | 1.33 | 0.09 | 15.32 | <0.001* | Season | 1.78 | 4 | 5.8 | 0.02* | 0.006 | 0.9 | 1205.9 |
|  |  |  |  |  |  | ONI | 0.00 | 5 | 0.0 | 0.88 |  |  |  |
| ONI | (Intercept) | 1.31 | 0.08 | 15.56 | <0.001* | ONI | 0.02 | 5 | 0.02 | 0.31 | 0.000 | 0.0 | 1207.3 |
| Roost | (Intercept) | 1.07 | 0.24 | 4.47 | <0.001* | Season | 1.75 | 4 | 6.53 | 0.02* | 0.018 | 2.26 | 1203.2 |
|  |  |  |  |  |  | Roost | 2.27 | 5 | 9.68 | 0.004* |  |  |  |
| Host age | (Intercept) | 1.58 | 0.11 | 14.72 | <0.001* | Season | 1.04 | 4 | 1.85 | 0.16 | 0.048 | 4.33 | 1190.7 |
|  | Juvenile | -1.22 | 0.21 | -5.86 | <0.001* |  |  |  |  |  |  |  |  |
|  | Subadult | -0.26 | 0.29 | -0.89 | 0.37 |  |  |  |  |  |  |  |  |
| Host sex | (Intercept) | 1.28 | 0.12 | 10.79 | <0.001* | Season | 1.76 | 4 | 5.79 | 0.02* | 0.006 | 0.91 | 1204.1 |
|  | Male | 0.07 | 0.17 | 0.43 | 0.67 |  |  |  |  |  |  |  |  |
| Host reproductive status (female) | (Intercept) | 1.28 | 0.26 | 4.92 | <0.001* | Season | 2.05 | 4 | 6.74 | 0.02* | 0.028 | 4.12 | 611.2 |
|  | Non-repro | 0.51 | 0.59 | 0.86 | 0.39 |  |  |  |  |  |  |  |  |
|  | Pregnant | 0.24 | 0.42 | 0.56 | 0.57 |  |  |  |  |  |  |  |  |
|  | Repro | 0.83 | 0.57 | 1.46 | 0.14 |  |  |  |  |  |  |  |  |
|  | Juvenile | -0.95 | 0.57 | -1.66 | 0.09 |  |  |  |  |  |  |  |  |
|  | Subadult | -0.49 | 0.56 | -0.88 | 0.38 |  |  |  |  |  |  |  |  |
| Host reproductive status (male) | (Intercept) | 3.74 | 1.47 | 2.53 | 0.01* | Season | 0.41 | 4 | 0.51 | 0.28 | 0.093 | 8.25 | 569.9 |
|  | Repro | -2.08 | 1.47 | -1.42 | 0.16 |  |  |  |  |  |  |  |  |
|  | Juvenile | -3.86 | 1.50 | -2.57 | 0.01* |  |  |  |  |  |  |  |  |
|  | Subadult | -1.75 | 1.36 | -1.28 | 0.20 |  |  |  |  |  |  |  |  |
| Host species | (Intercept) | 1.32 | 0.09 | 15.28 | <0.001* | Season | 1.78 | 4 | 5.96 | 0.02* | 0.01 | 1.23 | 1203.2 |
|  | GHFF | -2.08 | 1.23 | -1.69 | 0.09 |  |  |  |  |  |  |  |  |
| Host condition | (Intercept) | 1.33 | 0.09 | 15.02 | <0.001* | Season | 1.88 | 4 | 6.89 | 0.01* | 0.016 | 1.77 | 1201.2 |
|  |  |  |  |  |  | Host condition | 0.88 | 9 | 6.26 | 0.007* |  |  |  |
| *Borrelia GAM* |  |  |  |  |  |  |  |  |  |  |  |  |  |
| Season | (Intercept) | -3.88 | 0.24 | -15.83 | <0.001* | Season | 0.00 | 4 | 0 | 0.41 | 0.000 | 0.14 | 855.52 |
|  |  |  |  |  |  | ONI | 0.11 | 5 | 0.13 | 0.29 |  |  |  |
| *Bartonella* GLM | (Intercept) | 1.25 | 0.15 | 8.23 | <0.001* |  |  |  |  |  |  |  |  |
|  | Nycteribiid positive | 0.08 | 0.18 | 0.47 | 0.64 |  |  |  |  |  |  |  |  |
| *Borrelia* GLM | (Intercept) | -5.52 | 1.00 | -5.51 | <0.001* |  |  |  |  |  |  |  |  |
|  | Nycteribiid positive | 1.96 | 1.03 | 1.89 | 0.06 |  |  |  |  |  |  |  |  |

*Statistically significant (p < 0.05).

Table S3: PCA loadings and eigenvalues as proportion of variation from PCA of session abiotic variables.

| Variable | PC1 | PC2 | PC3 | PC4 | PC5 |
| --- | --- | --- | --- | --- | --- |
| Monthly mean temperature | -0.556 | -0.214 | -0.129 | 0.711 | -0.351 |
| Monthly mean humidity | -0.14 | 0.754 | -0.273 | 0.311 | 0.491 |
| Monthly mean solar radiation | -0.454 | -0.506 | -0.102 | -0.178 | 0.704 |
| Monthly total evaporation | 0.529 | -0.245 | 0.404 | 0.598 | 0.374 |
| Monthly total precipitation | -0.431 | 0.263 | 0.858 | -0.097 | 0.01 |
| Eigenvalue as a proportion of variation | 0.552 | 0.311 | 0.102 | 0.024 | 0.009 |

Greater than 80% variation described by PC1 and PC2. PC1 describes temperature and evaporation primarily (based on a threshold of 0.5) and PC2 mostly describes humidity.

**Fig. S1** Proportion of female nycteribiids collected per session and binomial 95% confidence interval. Each point is coloured by the roost location. Dark grey shading from 1^st^ June till 31^st^ August indicates the winter period; Light grey shading from 1^st^ September till 30^th^ November indicates spring.

**Fig. S2** Nycteribiid median intensity and 95% confidence interval coloured by roost location. Each observation represents a single catching session. Dark grey shading from 1^st^ June till 31^st^ August indicates the winter period; Light grey shading from 1^st^ September till 30^th^ November indicates spring.

**Fig. S3** Model based conditional predictions and 95% confidence intervals of probability of nycteribiid parasitism by ONI anomaly (°C). Points are prevalence for each session plotted by ONI anomaly.

**Fig. S4** Session prevalence by median intensity coloured by aggregation. As prevalence and intensity increase, aggregation decreases.

**Fig. S5** PCA biplot of abiotic variables for each session. Points are coloured by the roost location and sized by session prevalence. Ellipse drawn for Redcliffe (orange) and Toowoomba (blue) sessions depicts 68% of the Normal distribution (1 standard deviation) for each group’s mean. Humidity has the greatest loading for PC2 and is the gradient along which Toowoomba and Redcliffe sessions diverge most. This is supported by PERMANOVA of PC2 (F_8,32_ = 4.16, R^2^ = 0.51, p = 0.004).

**Fig. S6** Model based conditional predictions and 95% confidence intervals of the probability of nycteribiid parasitism by reproductive status in male bats (Non-rep. = non-reproductive, Rep. = reproductive) conditional on a mean month of 6.86 (25th June) and age fixed as adult.

**Fig. S7** Model based conditional predictions and 95% confidence intervals of the probability of nycteribiid parasitism by host species (BFF = black flying fox, *Pteropus alecto*; GHFF = grey-headed flying fox, *P. poliocephalus*) conditional on a mean month of 6.83 (25th June).

**Fig. S8** Model based conditional predictions and 95% confidence intervals of the probability of nycteribiid parasitism by host condition (standardised body weight) conditional on a mean month of 6.83 (25th June).

**Fig. S9** Median-joining haplotype network of *Bartonella* and *Borrelia* sequences obtained from Australian black flying foxes (*Pteropus alecto*). The networks were produced from alignments of 244 *Bartonella* citrate synthase gene (gltA) sequences (377 bp) and 11 *Borrelia* flagellin gene (flaB) sequences (502 bp). In the network, the size of coloured circles represents total sequences within a haplotype. Small solid black circles between nodes indicate median vectors. Hash marks between nodes indicate the number of nucleotide substitutions separating haplotypes.
